# A call to action for delirium research: Meta-analysis and regression of delirium associated mortality

**DOI:** 10.1186/s12877-020-01723-4

**Published:** 2020-09-07

**Authors:** May Zin Aung Thein, Jarett V. Pereira, Anita Nitchingham, Gideon A. Caplan

**Affiliations:** 1 Faculty of Medicine, University of New South Wales, Edmund Blackett Building, Prince of Wales Hospital, Barker Street, Randwick, NSW 2031 Australia; 2 Department of Geriatric Medicine, Prince of Wales Hospital, Prince of Wales Clinical School University of New South Wales, Sydney, Australia

**Keywords:** Delirium, Acute confusion, Death, Mortality, Prognosis

## Abstract

**Background:**

Delirium is an extremely common hospital complication. No study to date has assessed whether a priori defined covariates; type of hospital setting and year of study publication, influence the relationship between delirium and mortality. This is also the first study to examine the longitudinal trend of delirium-associated mortality over recent decades, to analyse the trajectory of our efforts in combating this disease.

**Methods:**

MEDLINE, EMBASE and PsycINFO, were searched from January 1981 to May 2018 for English-language primary articles. Rigorous title and abstract screen and full-text screen were conducted independently by two reviewers. This paper adhered to MOOSE guidelines. Data was extracted independently by one reviewer using standardised data-collection sheets, with a separate reviewer verifying for accuracy. The quality of included studies was assessed using the Newcastle-Ottawa Quality Assessment Scale. Unadjusted effect sizes and event counts were analysed with a random effects model in primary meta-analysis and meta-regression, whereas a mixed effect model was used in secondary sub-group analysis. Mortality data at longest follow-up and cumulative mortality (hospital mortality combined with mortality at longest follow-up) data were analysed.

**Results:**

As part of a larger project, 446 of 6790 articles were retrieved, including 71 studies that measured mortality. Our results demonstrate that elderly inpatients with delirium had significantly greater odds of mortality (OR 3.18 [95%CI: 2.73, 3.70]) compared to non-delirious controls. Patients with delirium in the ICU had the highest odds for mortality (OR: 7.09 [95%CI: 3.60, 14.0]); double the risk compared to the average. Curiously, despite advancements in delirium research, delirium associated in-hospital odds of mortality has not changed in 30 years.

**Conclusion:**

This is the largest meta-analysis to confirm the association between delirium and mortality, in older (age ≥ 65) hospital inpatients.

The current meta-analysis highlights the significant odds of mortality after an episode of delirium, and these odds are much higher for ICU patients. However, in contrast to other medical conditions that have seen a decrease in associated mortality over the past few decades, delirium associated mortality remains unchanged. These findings underscore the urgent need for better delirium treatments.

**PROSPERO Registration Number**: CRD42018098627, https://www.crd.york.ac.uk/prospero/display_record.php?RecordID=98627

## Background

Delirium is an acute state of confusion characterised by inattention, cognitive dysfunction and a fluctuating course associated with a medical cause [1], clinically diagnosed with the aid of delirium diagnostic tools such as the Confusion Assessment Method (CAM) [2]. Delirium is a significant clinical problem faced by clinicians [3], with a prevalence of around 22.9% [4] across acute and rehabilitation hospital wards. Meanwhile, an astonishingly high incidence of up to 89% of delirium occurs in the intensive care setting [5]. Overall, it is thought to affect up to half of older (aged ≥65 years) hospital inpatients [6]. Despite being of such importance, delirium remains poorly understood, and there are limited effective management options. There is scarce research on the pharmaceutical interventions for delirium [7], while non-pharmacological delirium interventions have risen in popularity. Multi-component and some single-component non-pharmacological interventions have demonstrated efficacy in prevention, but not in the treatment of delirium in patients > 60 years old [8]. Furthermore, the incidence and duration of delirium in critically ill patients were not reduced by non-pharmacological interventions [9]. This lack of effective treatment is drastically different from other diseases that have similar prevalence rates.

Therefore, there is a pressing need to evaluate the effect of delirium on the simplest and most significant marker of health - mortality.

In 1987, delirium was still defined as a transient disorder [10]. We now understand that delirium is associated with several negative sequelae. A meta-analysis in 2010 established that delirium in older people was associated with increased mortality, institutionalisation and dementia [11]. This underlines the importance of delirium recognition and treatment, as failure to diagnose delirium may have devastating consequences.

Recent studies examining the effect of delirium on mortality across various settings have had inconsistent findings [12–16]. Newer meta-analyses on this topic have been conducted in 2013 and 2015, both focused on critically ill populations [17, 18], while one in 2017 focused specifically on postoperative delirium [19]. Therefore, our current clinical practice is informed by studies with small sample size and isolated surgical or critically ill populations. An updated and statistically robust review is required to make a public health level impact statement that will change the way delirium is understood. Additionally, with the advent of multicomponent interventions, delirium tools, and efforts to increase delirium awareness, reviewing the trend of delirium-associated mortality over time would be a valuable reflection of the medical community’s efforts in combating delirium. Therefore, a rigorous quantitative review of the prognosis associated with delirium is crucial and timely.

Our study aims to explore the relationship between delirium and its sequelae, in older (≥ 65 years old) hospital inpatients, as well as the prognosis of delirium for patients in different settings (e.g. medical, surgical).

## Methods

This paper is part of a larger project examining the association between delirium and adverse outcomes in older (≥ 65 years old) hospital inpatients, compared to non-delirious controls using a meta-analysis and meta-regression approach. We endeavoured to systematically review unadjusted effect sizes and raw event counts to produce a comprehensive and summarised picture of the adverse consequences of delirium. Since adjustment varies from study to study, examining unadjusted data allows the study of raw mortality rates over time.

The focus of this paper will be on

1. Primary meta-analysis of the effect of delirium on mortality, as an adverse outcome, in older hospital inpatients.
2. Secondary subgroup meta-analysis comparing the effect of delirium on mortality within different settings (medical, surgical).
3. Secondary meta-regression analysis of the effect of the association between delirium and mortality, over time (year of publication).

### Data sources and search strategy

This project adhered to the Meta-analyses Of Observational Studies in Epidemiology (MOOSE) guidelines. A comprehensive and systematic literature search of EMBASE, MEDLINE and PsycINFO was developed with a librarian and with a specialist of geriatric medicine. Search terms including, “delirium”, “acute confusion”, “prognos* (prognosis/prognoses/prognostic) OR course* OR sequalae or outcome*”, “mortality”, were combined with appropriate Boolean operators, while considering the alternative spellings used in different countries (Supplementary Table S1). Primary articles written in English and published from January 1981 to 2018 were considered, while conference abstracts, posters and non-full text articles were excluded. The year 1981 was chosen as a starting point because the Diagnostic and Statistical Manual of Mental Disorders-3, which provided the first formal differentiation between dementia and delirium, was first published in 1980. Titles and abstracts were retrieved throughout 26th–29th May 2018. The protocol was successfully registered in the PROSPERO registry on 26th June 2018, (Registration Number: CRD42018098627) and the full search strategy is documented in Supplementary Table S1.

### Selection criteria

A title and abstract screen and a full text screen were conducted independently by the study investigators (MZAT and JP), and any disagreements were resolved through consultation with a co-author (GC). Both reviewers adhered to the inclusion and exclusion criteria (Supplementary Table S2). Random samples of 100 articles were trialled until inter-rater agreement reached 99%, and Kappa value 0.85, before commencing on the actual screening process. The inclusion and exclusion criteria were refined between title/ abstract screen, and full-text screen (specifics in Supplementary Table S2), to limit heterogeneity of studies without compromising the quantity of data that could be gathered.

**Fig. 1:**
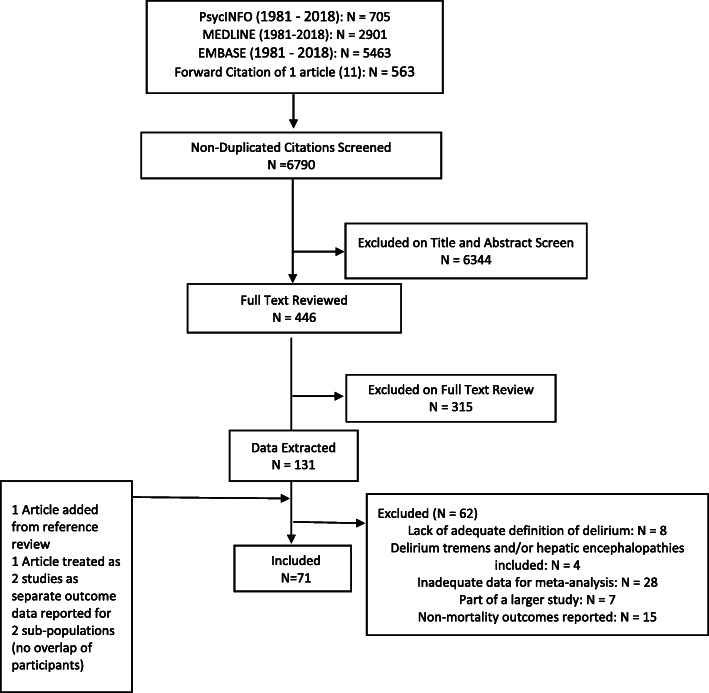
PRISMA flow chart describing process of article search and selection

After eliminating duplicates using Rayyan software [20], 6790 entries were reviewed for the three adverse outcomes of mortality, nursing home placement and dementia. Articles with the same title, author name and abstract content but a different journal title and/or issue number were also considered duplicates, and studies investigating delirium intervention were excluded. Hand searching in terms of a forward citation of a similar article [11] published in 2010 was also conducted to expand the breadth of this search. This process is documented in the PRISMA flow-chart (Fig. 1).

### Data extraction

A standardised data-collection sheet was used to extract data, including but not limited to the following variables: study design, criteria for diagnosis of delirium, number of deaths, time of longest follow-up for mortality outcome (Supplementary Table S3). Characteristics of included studies are recorded in Additional file 1: Table 4. Studies were given a unique identifying number, which appears before the primary author on the forest plots. Every possible effort was undertaken to obtain included articles by contacting corresponding authors.

One reviewer independently extracted the data, while another reviewer checked the data-extraction sheet for errors and ambiguous recordings. All disagreements were resolved by discussion and consultation with GC. In cases where several studies were part of the same larger study, data was obtained from the study with the largest sample size. For studies that measured outcomes at various time points, data at the longest follow-up was used. Cumulative mortality (hospital mortality combined with mortality at longest follow-up) data were analysed.

### Risk of bias and quality assessment

Quality assessment was conducted using the Newcastle-Ottawa Quality Assessment Scale (NOS) and each study was given an overall rating. All included articles were observational cohort studies. In accordance with previous literature that used the NOS, a summary score estimate of 0–3 stars reflected low quality, 4–6 stars moderate quality and 7–9 stars indicated high quality [21]. Articles with an overall rating of < 5 stars were excluded from our analysis.

### Statistical analysis

All statistical analyses were conducted with Comprehensive Meta-Analysis V3 software. For primary analysis, the overall effect size was calculated using either reported raw data of event counts or reported unadjusted effect sizes with their 95% confidence intervals (CI). All analyses were conducted using random effects models to produce an overall odds ratio (OR) as the principal summary measure. A random effects model was used as the assumption of true differences in exposure effect between studies, and its estimates of average exposure effect [22] was more conservative and realistic. For secondary analysis, studies were sub-grouped according to setting, as a categorical moderator and analysed for overall effect size within each sub-group.

Meta-regression was used to explore if the following a priori defined covariates accounted for heterogeneity of study-level effect sizes [23]:

1. Setting (categorical variable)
2. Year of publication (continuous variable)
  - Not all studies reported their year of study, therefore year of publication was adopted as a homogenous time measurement. This should not affect our analysis as this paper is interested in observing temporal trends of study results, instead of study results relative to an absolute time point.

These covariates were chosen for their high variability between, rather than within, studies [23]. Assessment and interpretation of heterogeneity are crucial in understanding meta-analytic results. Heterogeneity of effect sizes were analysed by I^2^ analysis: 25% was low heterogeneity; 50% moderate, and 75% considered high heterogeneity [24]. A meta-regression analysis will explore the reasons why the overall effect size obtained from the meta-analysis did not correspond to each study-level effect size. A random effects meta-regression was undertaken, because not all the heterogeneity can be accounted for by our meta-regression, with the inevitable existence of “residual heterogeneity” [23].

Sensitivity analyses used to test the robustness of the results included the secondary analyses on setting, and running the analysis with a fixed effects model (to determine if a similar result to the random effects model was produced). Observational studies, which are used in this study, are more susceptible to publication bias which can jeopardise the validity of meta-analyses [25]. Therefore, publication bias was rigorously assessed with several tools, including the Classic Fail-safe N test, which suggests the number of unpublished null result studies required to bring the *p*-value to > 0.05. Using a funnel plot, publication bias is suggested when the plot appears asymmetrical [25]. This subjective visual method was complemented with an objective Egger’s test intercept value. The intercept value is a quantitative measure of asymmetry which deviates further from zero with greater asymmetry [26]. A *p* value of < 0.05 was considered statistically significant.

## Results

### Primary analysis of mortality associated with delirium

**Fig. 2:**
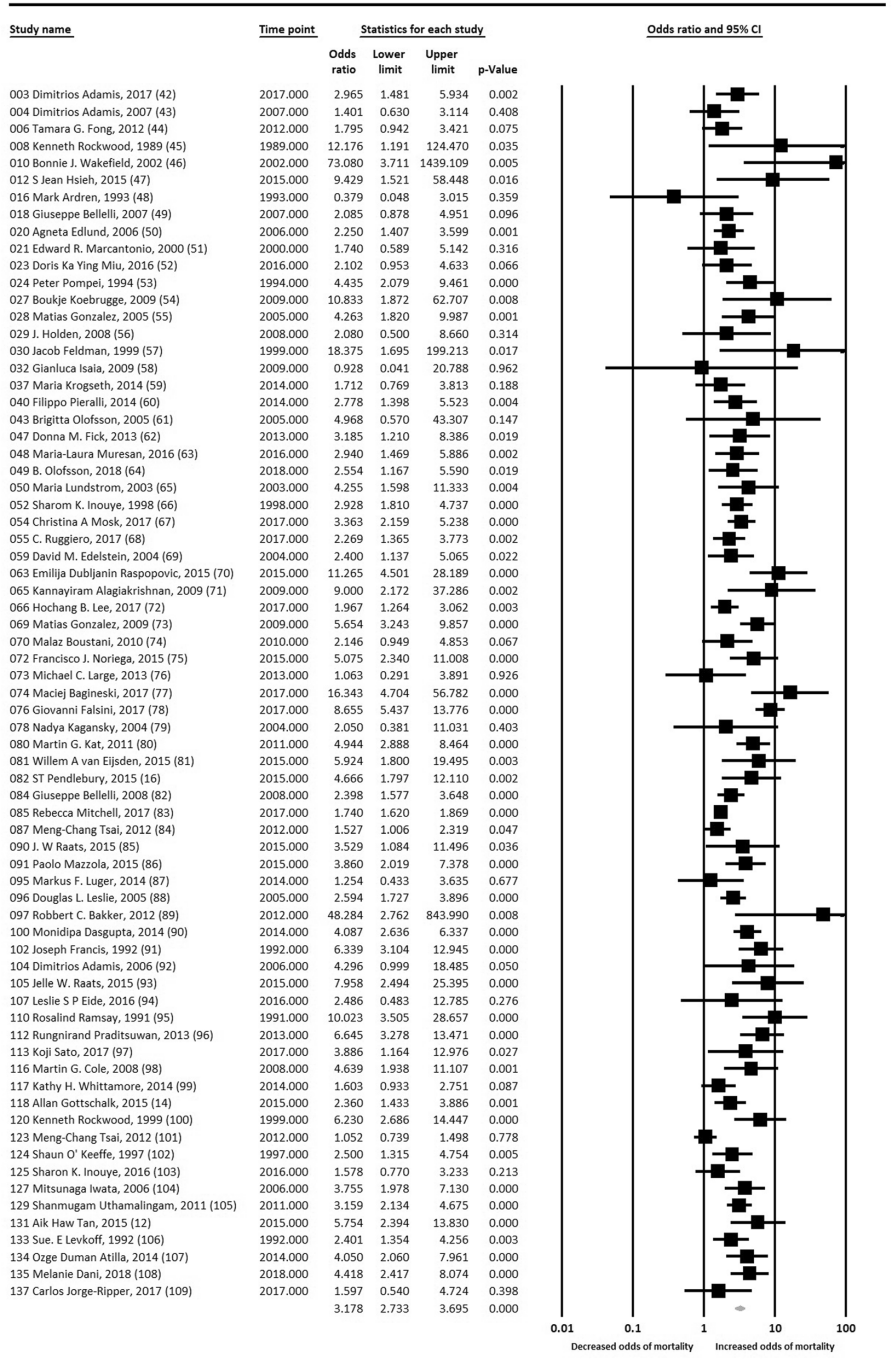
Primary analysis of unadjusted data from 71 studies ([42–109]). Odds Ratio of > 1 indicates greater odds of mortality in patients who experienced delirium. Studies named according to unique identification number and name of primary author. Time point indicates the year of publication. Lower limit and upper limit mark the confidence intervals (CI) of the odds ratio. (Please note: software does not allow us to remove column “Time point”)

A total of 71 studies were included in the primary analysis, amounting to > 49,566 participants (some studies provided OR without specifying the number of patients). Older patients who had an episode of delirium had significantly greater odds of mortality (OR 3.18, 95% CI: 2.73, 3.70) compared to non-delirious controls (Fig. 2). There was significant statistical heterogeneity between the included studies (I^2^ = 73.9%, *P* < 0.001), that will be analysed using a meta-regression approach in subsequent sections.

Sensitivity analysis with fixed effects model showed that delirium was significantly associated with mortality regardless of the type of model used (OR 2.17, 95% CI: 2.10, 2.30). On visual inspection of the funnel plot, asymmetry was observed, and presence of publication bias was suggested on Egger’s Test of the Intercept (1.63, 95% CI: 1.13, 2.13; *p* < 0.001). However, the classic fail-safe N indicated that 12,838 “null” studies would be required for the *P*-value to > 0.050. Further, Duval and Tweedie’s trim-and-fill analysis, which recomputes the overall OR taking into account articles that would have been omitted due to publication bias, produced an imputed point estimate OR of 2.10 (95% CI: 1.80, 2.46). The results of both tests indicate low risk of publication bias.

### Secondary sub-group analysis of mortality in various settings

Secondary analysis using a random effects model was conducted with studies stratified according to hospital setting: intensive care unit (ICU), medical (e.g. general medical or acute wards), surgical, mixed (e.g. acute and post-acute wards, surgical and medical wards) or post-acute (e.g. rehabilitation and/or psychiatry).

Patients who experienced delirium in different settings had different odds for mortality: OR: 7.09 (95% CI: 3.60, 14.0) in the ICU setting, OR: 3.64 (95% CI: 2.99, 4.44) in the medical setting, OR: 3.00 in the surgical setting (95% CI: 2.33, 3.88), OR: 2.98 in the mixed setting (95% CI: 2.30,3.86) and lastly OR: 1.67 in the post-acute setting (95% CI: 1.17, 2.39).

### Meta- regression analysis of mortality in various settings

**Fig. 3:**
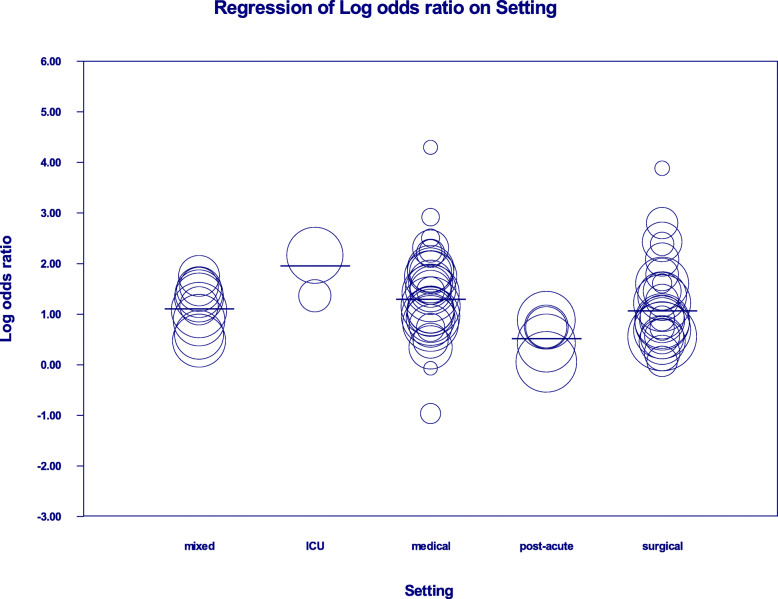
Meta-regression analysis using setting (ICU, mixed, medical, post-acute, surgical) as a covariate to measure the effect of delirium on mortality. Each circle represents one study, and the size of the circles are proportional to the study size. Overall effect size in each group indicated as a horizontal line

A meta-regression with setting as a covariate in a random effects model was subsequently carried out. With an R^2^ of 0.41, and a *p*-value of 0.006, different settings appear to account for a significant portion of the heterogeneity observed in the overall primary analysis (Fig. 3).

An exploratory secondary analysis used mixed setting as a reference point (intercept). There was no significant difference in delirium associated mortality between the medical, surgical and mixed settings (R^2^ of 0.05, *P* value of 0.24).

In a follow-up analysis, we grouped medical, surgical and mixed settings together, and compared this to the ICU setting. There was a trend towards higher mortality in ICU (R^2^ of 0.19, *P*-value of 0.06). When post-acute setting was compared to the combined medical, surgical and mixed setting, there was significant heterogeneity, indicating lower mortality in post-acute settings (R^2^ of 0.27, P- value of 0.004).

### Meta-regression analysis of mortality over the years

**Fig. 4:**
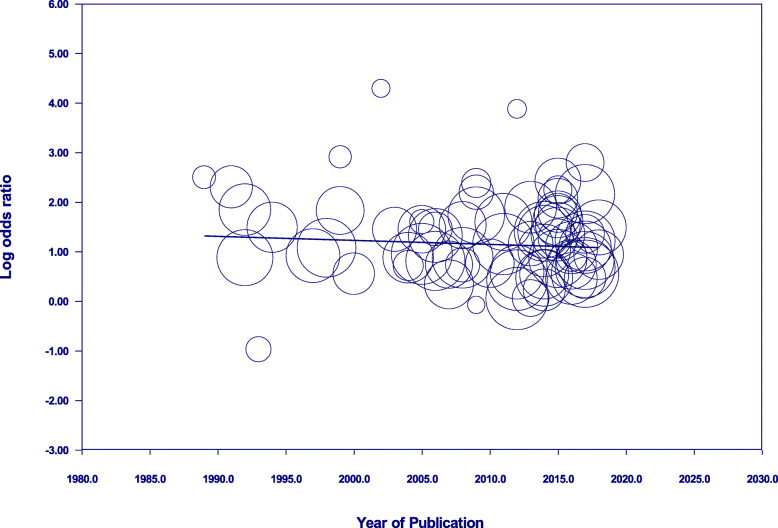
Meta-regression analysis using year of publication (continuous value) as a covariate to measure the effect of delirium on mortality. Each circle represents one study, and the size of the circles are proportional to the study size

Year of publication did not account for significant heterogeneity between effect sizes across all our included studies (R^2^ of 0.00, *P*-value of 0.44) (Fig. 4).

When the medical (R^2^ 0.00, P 0.40), surgical (R^2^ 0.00, P 0.91) and mixed (R^2^ of 0.00, P 0.99) settings were analysed in isolation, year of publication did not account for the heterogeneity observed in delirium associated mortality. Since there was no significant heterogeneity in the above three settings, they were combined in subsequent temporal analysis (R^2^ 0.03, P 0.30), but remained unaccountable for heterogeneity in delirium mortality. A separate analysis was conducted for the post-acute setting (R^2^ 0.06, P 0.51), whereas the ICU was excluded because it only had two studies published in the setting, both in the same year. The regression analyses demonstrate that year of publication does not influence delirium associated mortality, regardless of setting.

## Discussion

This is the first study to find that delirium related mortality has not decreased in the past 30 years. It is also the largest meta-analysis to-date analysing the relationship between delirium and mortality. Our findings show that delirium is associated with increased risk of all-cause mortality in elderly hospitalised patients compared to sick inpatients without delirium, which emphasises that delirium is a serious medical condition requiring immediate investigation and management. This result also serves as an important call to action, for increased efforts to improve implementation of delirium management in clinical practice, and more research into delirium treatment and prevention.

### Association between delirium and mortality

The exact nature of the relationship between delirium and death remains unknown, and several theories have been proposed to explain this phenomenon. These theories are likely overlapping and complementary, interacting to contribute to mortality [27]. Delirium may directly cause brain damage through a combination of mechanisms including neuroinflammation, neurotransmitter dysregulation [28] and disrupted cerebral metabolism [29]. These processes cause neuronal dysfunction and eventually lead to irreversible neuronal destruction, potentially explaining why the effects of delirium often persist long after each episode. Delirium may also cause death indirectly through various hospital-acquired complications. These are sequelae of delirium, such as increased risk of falls, aspiration pneumonia, pressure ulcers and administration of restraining devices; all of which result in increased morbidity and mortality [30]. That delirium also increases hospital length of stay and ICU length of stay [18], further compounds the problem by increasing the risk of acquiring in-hospital complications. Further research into the pathophysiology of delirium is needed to fully understand why delirium leads to adverse consequences.

### Association between delirium related mortality and clinical setting

There was considerable statistical heterogeneity between studies for the outcome of mortality, according to the I^2^ analysis. Our meta-regression showed that only setting, but not year of publication, accounts for this heterogeneity. Therefore, the residual heterogeneity may be explained by other covariates such as age, baseline cognitive state and effectiveness of therapeutic interventions, all of which were difficult to incorporate into this study because of variable reporting.

Subgroup analysis focusing on the effect of study setting on delirium-associated mortality found greater mortality in the acute setting compared to the post-acute setting. This is the first meta-analysis to compare delirium-associated mortality between different hospital settings. The odds ratio was greater in the acute setting, especially ICU, compared to the post-acute setting. A likely explanation for the difference in effect size is the greater severity of illness in ICU. An alternative explanation could be that ICU delirium may mediate the effect of increased mortality indirectly, through increasing length of stay which in turn, prolongs exposure of critically ill patients to nosocomial complications [31]. Our findings highlight that ICU delirium is a potentially fatal complication. However, only 40% of health professionals consistently screen for ICU delirium [32], suggesting that there is scope for improved detection and effective treatment of delirium in ICU and acute settings to improve outcomes. Furthermore, the variation in delirium mortality across different settings suggests that tailored management for patients with delirium may be required.

### Delirium associated mortality over the years

With the advent of technology and the progressive developments in delirium research, delirium outcomes were compared temporally. We made a novel discovery that delirium associated mortality had not changed over the past 30 years. This was in contrast to the decline in cardiovascular mortality observed in the past four decades [33], as well as the steady drop of approximately 1.5% per year in cancer mortality over the past two decades [34]. While pursuing an explanation for such a phenomenon, it was found that neither antipsychotic pharmacological therapy [35] nor non-pharmacological [36] interventions showed any benefit in terms of decreasing mortality. Greater awareness of delirium over the past three decades would have been expected to result in increasing diagnostic rates and lower mortality simply due to greater detection of milder cases. However, the fact that mortality has not changed suggests that we must consider novel research and treatment options. An intriguing finding that delirium incidence is reduced with alternate treatment settings in randomised and non-randomised controlled trials of Hospital in the Home [37] and that treatment in those settings reduces mortality by 19% in the meta-analysis [38] suggests another possible option for reducing delirium mortality.

### Strengths and limitations

The limitations in this study stem from the large number of articles included, and therefore the unavoidable diversity in methodology. For instance, censored data could not be removed from the total sample size in some articles, while they were deducted in others. Further, while we aimed to use cumulative mortality data, some articles excluded in-hospital deaths from deaths at follow-up. Additionally, different diagnostic criteria of delirium may have also contributed to the heterogeneity of results. Another important confounder is the different rates of antipsychotic prescription between countries. For instance, the rate of prescription was 85.5% in Thailand [39], compared to 66% prescription rate for severe delirium in Melbourne [40]. Considering that antipsychotics like haloperidol have been associated with 73% increased mortality compared to placebo (hazard ratio,1.73; 95% CI, 1.20–2.50; *P* = .003) [41], variation in prescription rates across the world could influence delirium associated mortality.

We could not do meta-regression on age as a covariate because many studies lacked data on mean age. Similarly, we acknowledge that we have not been able to evaluate the baseline characteristics (e.g. cognitive status, age, gender, and medical comorbidities) despite attempts to gather this information. There was significant missing information across different studies, making it unsuitable for comparative analysis.

Lastly, we would like to emphasize that while the ICU setting yield the highest overall OR of 7.09 (95% CI: 3.60, 14.0), this is an analysis of merely 2 studies. Future studies will therefore require more robust data, and in higher quantities, to support this finding.

Nonetheless, these limitations should not affect the strength of our current results, as we have included many high-quality studies with a large sample size. Non-English articles and data were not included due to practical limitations. At a review level, it must be acknowledged that no screening, retrieval, data-extraction or quality-assessment process is perfect, although the utmost effort had been made to ensure accuracy in conducting the study.

Our study also has many strengths. Our analyses with an enormous sample size give the study more statistical power and allows for the discovery of previously hidden trends. The most appropriate analytic models were also chosen. Although this study did not include grey literature, publication bias was rigorously analysed and did not appear to influence the reported associations. Therefore, it is unlikely that the results of this meta-analysis will be contradicted by larger trials. The high quality of included studies was ensured by strict inclusion and exclusion criteria as well as a comprehensive NOS assessment. For instance, studies that did not diagnose delirium using a validated delirium instrument, and articles with ambiguity in reported data were excluded.

## Conclusion

Our findings challenge the archaic misconception that delirium is a transient and reversible disorder. The results are largely generalisable to the older inpatient population as studies over a variety of settings and countries were analysed; this may therefore encourage policy makers to design delirium management protocols. Our novel findings from stratified setting analysis may act as evidence for the need to carefully allocate resources to different departments for delirium management. We must recognise that delirium mortality has not decreased over time as expected. There is publication bias towards studies that support the positive results of medical advancements. However, studies, like ours that show negative results are equally important, as it beckons re-evaluation of our efforts in understanding and treating this disturbingly prevalent condition. Therefore, this study also aims to raise awareness regarding the urgency and gravity of delirium sequelae, and the need for increased research into the identification, pathophysiology and treatment of delirium, because delirium related mortality has not improved over the past 30 years, whereas outcomes of other disease have.

## Supplementary information

**Additional file 1: Table S1.** Search Strategy. **Table S2.** Inclusion and Exclusion Criteria. **Table S3.** Types of Data Extracted from Each Article. **Table S4.** Characteristics of Included Studies. **Figure S1.** Secondary Sub Group Analysis of Delirium and Mortality According to Setting. a. A secondary analysis of all 71 included studies, sub-grouped into their clinical settings (ICU, medical, post-acute, surgical or mixed), showing that the highest odds of mortality occurred in the ICU population OR 7.09 (95% CI: 3.60, 14.0).

## Data Availability

**PROSPERO Registration Number**: CRD42018098627, https://www.crd.york.ac.uk/prospero/display_record.php?RecordID=98627 Additional files – supplementary material.

## Abbreviations

CAM: Confusion Assessment Method
MOOSE: Meta-analyses Of Observational Studies in Epidemiology
NOS: Newcastle-Ottawa Quality Assessment Scale
CI: Confidence intervals
OR: Odds ratio
ICU: Intensive care unit
