# Supplementary material for: A call to action for delirium research: Meta-analysis and regression of delirium associated mortality
Source: BMC Geriatr. 2020 Sep 7;20:325. doi: 10.1186/s12877-020-01723-4 (PMC7487610; doi:10.1186/s12877-020-01723-4)
Supplement: Supplementary file 1 — Additional file 1: Table S1. Search Strategy. Table S2. Inclusion and Exclusion Criteria. Table S3. Types of Data Extracted from Each Article. Table S4. Characteristics of Included Studies. Figure S1. Secondary Sub Group Analysis of Delirium and Mortality According to Setting. a. A secondary analysis of all 71 included studies, sub-grouped into their clinical settings (ICU, medical, post-acute, surgical or mixed), showing that the highest odds of mortality occurred in the ICU population OR 7.09 (95% CI: 3.60, 14.0). [file 12877_2020_1723_MOESM1_ESM.docx]

This document includes:

1. Supplementary Table S1: Search Strategy
2. Supplementary Table S2: Inclusion and Exclusion Criteria
3. Supplementary Table S3: Types of Data Extracted from Each Article
4. Supplementary Table S4: Characteristics of Included Studies
5. Supplementary Figure S1: Secondary Sub Group Analysis of Delirium and Mortality According to Setting

**Supplementary Table S1: Search Strategy**

| Data Base | Search No. | Search Strategy | Hits |
| --- | --- | --- | --- |
| MEDLINE  (26/05/2018) | 1 | delirium.mp. or exp DELIRIUM/ | 13242 |
|  | 2 | acute confusion*.mp. | 773 |
|  | 3 | 1 or 2 | 13797 |
|  | 4 | exp MORTALITY/ | 342787 |
|  | 5 | exp DEATH/ | 137768 |
|  | 6 | 4 or 5 | 466504 |
|  | 7 | exp Nursing Homes/ or exp Institutionalization/ | 43864 |
|  | 8 | institution*.mp. | 229833 |
|  | 9 | Homes for the Aged/ | 12869 |
|  | 10 | 7 or 8 or 9 | 268692 |
|  | 11 | exp DEMENTIA/ | 146823 |
|  | 12 | exp Cognition Disorders/ | 81289 |
|  | 13 | 11 or 12 | 201473 |
|  | 14 | (prognos* or course* or sequalae or outcome*).mp. | 2728417 |
|  | 15 | 6 or 10 or 13 or 14 | 3314862 |
|  | 16 | 3 and 15 | 5661 |
|  | 17 | limit 16 to (english language and humans and yr="1981 - 2018" and "all aged (65 and over)") | 2901 |
| EMBASE  (29/05/2018) | 1 | delirium.mp. or exp delirium/ | 31084 |
|  | 2 | acute confusion*.mp. | 1619 |
|  | 3 | 1 or 2 | 32243 |
|  | 4 | exp mortality/ | 979455 |
|  | 5 | exp death/ | 720708 |
|  | 6 | 4 or 5 | 1541921 |
|  | 7 | nursing home/ | 50374 |
|  | 8 | exp institutionalization/ | 8509 |
|  | 9 | institution*.mp. | 397685 |
|  | 10 | home for the aged/ | 12317 |
|  | 11 | 7 or 8 or 9 or 10 | 447605 |
|  | 12 | exp cognitive defect/ | 430839 |
|  | 13 | (prognos* or course* or sequalae or outcome*).mp. | 4378798 |
|  | 14 | 6 or 11 or 12 or 13 | 5910541 |
|  | 15 | 3 and 14 | 17607 |
|  | 16 | limit 15 to (human and english language and yr="1981 - 2018" and aged <65+ years>) | 5463 |
| Psyc-INFO  (29/05/2018) | 1 | delirium/ | 3049 |
|  | 2 | acute confusion*.mp. | 354 |
|  | 3 | 1 or 2 | 3305 |
|  | 4 | "death and dying"/ or mortality rate/ | 33451 |
|  | 5 | exp Institutionalization/ or Nursing Homes/ | 37267 |
|  | 6 | Residential Care Institutions/ or Elder Care/ | 13748 |
|  | 7 | 5 or 6 | 49713 |
|  | 8 | exp DEMENTIA/ | 69470 |
|  | 9 | cognitive impairment/ | 32276 |
|  | 10 | 8 or 9 | 91962 |
|  | 11 | (prognos* or course* or sequalae or outcome*).mp. | 537664 |
|  | 12 | 4 or 7 or 10 or 11 | 678813 |
|  | 13 | 3 and 12 | 1432 |
|  | 14 | limit 13 to (human and english language and "380 aged <age 65 yrs and older>" and yr="1981 - 2018") | 705 |

**Supplementary Table S2: Inclusion and Exclusion Criteria**

| Original Inclusion and Exclusion Criteria |
| --- |
| Inclusion Criteria |
| 1. Mean age of population is ≥65 years 2. Population is in the hospital inpatient setting 3. Delirium diagnosed using a validated instrument (includes, but not restricted to DSM-4, DSM-5, CAM, 4-AT) 4. Quantitative data were provided to measure outcomes |
| Exclusion Criteria |
| 1. Reviews, conference abstracts, case studies and case series 2. Retrospective diagnosis of delirium 3. Patients recruited are only delirious patients, without recruitment of controls 4. Delirium tremens, delirium post-stroke, metabolic encephalopathies, and patients with prior focal brain pathology |
| Final Inclusion and Exclusion Criteria |
| Inclusion Criteria |
| 1. Population is ≥65 years 2. Population is in the hospital inpatient setting, including rehabilitation hospitals (excludes: nursing home, long term care residents, post-acute care, skilled nursing facilities) 3. Delirium diagnosed using a validated delirium diagnostic instrument (includes, but not restricted to DSM-4, DSM-5, CAM, 4-AT) 4. Quantitative data were provided to measure outcomes |
| Exclusion Criteria |
| 1. Reviews, case studies and case series 2. Any publication type that is not a full-text article (e.g. conference abstracts, poster abstracts, letter to editor etc.) 3. Retrospective diagnosis of delirium 4. Studies that only recruited delirious patients, without recruitment of control (non-delirious) patients. 5. Studies which specify that patients with delirium tremens and/or hepatic encephalopathies have been included 6. Patients specified to be in the palliative care, hospice, terminal cancer setting, or specifically mentioned to have brain metastasis |

CAM: Confusion Assessment Method, DSM: Diagnostic and Statistical Manual of Mental Disorders

**Supplementary Table S3: Types of Data Extracted from Each Article**

| Characteristics of Article | - Unique identification number of articles - Primary author - Publication year - Full Title - Outcomes measured in the article, and useful outcomes with data extracted for this study - Country - Part of a larger study (Yes or No) - Study design |
| --- | --- |
| Population | - Gender: Male (control population) (n/total control population) - Gender: Female (control population) (n/total control population) (%) - Gender: Male (delirium population) (n/total delirium population) (%) - Gender: Female (delirium population) (n/total delirium population) (%) - Total number of delirium patients - Total study population - Pre-existing dementia in this population (Qualitative data collected) - Pre-existing illness/setting (Qualitative data collected) - Any inclusion of patients with severe dementia (Qualitative data collected) - Criteria for diagnosis of delirium (Qualitative data collected) - Mean age of controls - Mean age of delirium patients - Mean age of population |
| Mortality | - Unique identification number of articles - Title of article - No. of in-hospital mortality in control patients (n) - Total number of control patients (n) - No. of in-hospital mortality in delirium patients (n) - Total number of delirious patients (n) - Longest time of follow-up - Cumulative with in-hospital death or not (measured by documenting if denominator has changed from original) - Mortality for longest time of follow-up in control population - Total number of control population - Mortality for longest time of follow-up in delirious patients - Total number of delirious patients - Odds Ratios (univariate or unadjusted), lower and upper limit of 95% CI - Odds Ratios (multivariate or adjusted), lower and upper limit of 95% CI - Hazard Ratios (univariate or unadjusted), lower and upper limit of 95% CI - Hazard Ratios (multivariate or adjusted), lower and upper limit of 95% CI - Risk Ratios (univariate or unadjusted), lower and upper limit of 95% CI - Risk Ratios (multivariate or adjusted), lower and upper limit of 95% CI - Variables adjusted for in effect size calculation (multivariate/adjusted effect sizes) |

**Supplementary Table S4: Characteristics of Included Studies**

| Number | Primary Author, Year of Publication, (reference) | Outcomes used | Country | Setting | Criteria for diagnosis of delirium | Delirium (N) | Total (N) | Mean age of population |
| --- | --- | --- | --- | --- | --- | --- | --- | --- |
| 003 | Dimitrios Adamis, 2017 ^42^ | M | Ireland | Acute medical admission | DSM-5, DSM-IV, CAM, DRS-R98 | 46 | 200 | 81.1 |
| 004 | Dimitrios Adamis, 2007 ^43^ | M | UK | Elderly medical unit | CAM & DRS | 47 | 164 | 84.6 |
| 006 | Tamara G. Fong, 2012 ^44^ | M | USA | Community-based | CAM | 194 | 367 | unspecified |
| 008 | Kenneth Rockwood, 1989 ^45^ | M | Canada | General medical services (excluding coronary care unit and ICU) | DSM-III | 20 | 80 | 76.8 |
| 010 | Bonnie J. Wakefield, 2002 ^46^ | M | USA | General medicine units (midwestern veterans affairs medical centre) | NEECHAM | 16 | 117 | 73 |
| 012 | S Jean Hsieh, 2015 ^47^ | M | USA | Non-ICU inpatient ward | CAM-ICU | 38 | 260 | unspecified |
| 016 | Mark Ardren, 1993 ^48^ | M, NH | UK | General medical wards | DSM-III-R (likely physician diagnosis) | 23 | 163 | 76.0 |
| 018 | Giuseppe Bellelli, 2007 ^49^ | M | Italy | Rehabilitation and Aged Care Unit (RACU) | CAM | 94 | 188 | unspecified |
| 020 | Agneta Edlund, 2006 ^50^ | M, NH | Sweden | 2/5 wards of the Department of General Internal Medicine | Confusion subscale of the Organic Brain Syndrome Scale + MMSE (replacing the disorientation subscale of OBS scale), DSM-IV | 125 | 400 | unspecified |
| 021 | Edward R. Marcantonio, 2000 ^51^ | M, NH | USA | Hip fracture repair surgery | Delirium Symptom Interview, CAM | 52 | 126 | 79 |
| 023 | Doris Ka Ying Miu, 2016 ^52^ | M | Hong Kong | Post-acute care hospital | Chinese CAM, DOS | 89 | 261 | 81.9 |
| 024 | Peter Pompei, 1994 ^53^ | M | USA | Medical and surgical wards | Screening instruments including CAM, and then independent assessment of clinician investigator using DSM-III-R | 64 | 432 | unspecified |
| 027 | Boukje Koebrugge, 2009 ^54^ | M | Netherlands | Elective abdominal surgery | DOS, DSM-IV | 17 | 71 | unspecified |
| 028 | Matias Gonzalez, 2005 ^55^ | M | Spain | Medical and traumatology wards | Spanish CAM, original and Spanish DRS | 58 | 149 | unspecified |
| 029 | J. Holden, 2008 ^56^ | M, NH | New Zealand | General medical and rehabilitation wards | CAM, MMSE, formal cognition testing, family and nursing observations | 54 | 162 | unspecified |
| 030 | Jacob Feldman, 1999 ^57^ | M | Israel | Acute care geriatric unit | Examination by an experienced geriatrician, CAM, DRS | 11 | 61 | unspecified |
| 032 | Gianluca Isaia, 2009 ^58^ | M | Italy | Traditional geriatric hospital ward | CAM, DRS | 10 | 60 | 84.7 |
| 037 | Maria Krogseth, 2014 ^59^ | M, NH | Norway | Hip fracture patients | Algorithm version of CAM, MMSE, orientation testing, clock drawing test, MDAS | 80 | 207 | 82.6 |
| 040 | Filippo Pieralli, 2014 ^60^ | M | Italy | Two internal medicine units | CAM in records | 110 | 434 | 81.8 |
| 043 | Birgitta Olofsson, 2005 ^61^ | M | Sweden | Femoral neck fracture treatment | Organic Brain Syndrome Scale, MMSE, nurse's and medical records, DSM-IV by physician's diagnosis | 38 | 61 | 82.6 |
| 047 | Donna M. Fick, 2013 ^62^ | M | USA | Community hospital | MMSE, CAM, DRS-revised-98, 24-hr period observations, nurse and family member interviews, chart review | 44 | 139 | 83 |
| 048 | Maria-Laura Muresan, 2016 ^63^ | M | Ireland | Acute medical admissions | CAM, DRS-R-98 | 46 | 199 | 81.13 |
| 049 | B. Olofsson, 2018 ^64^ | M, D | Northern Sweden | Femoral neck fracture | Modified OBS scale, DSM-IV-TR (by geriatrician retrospectively) | 76 | 135 | unspecified |
| 050 | Maria Lundstrom, 2003 ^65^ | D, M | Sweden | Operation for fractured neck of femur | OBS scale, DSM-IV | 30 | 78 | 79.1 |
| 052 | Sharon K. Inouye, 1998 ^66^ | M, NH | USA | Medical, surgical patients in acute care hospitals (excluding: intensive-care wards) | CAM | 90 | 727 | 78.9 |
| 054 | Christina A Mosk, 2017 ^67^ | M | Netherlands | Hip fracture surgery | Delirium Observational Screening Scale, physician involvement | 196 | 566 | unspecified |
| 055 | C. Ruggiero, 2017 ^68^ | M | Italy | Femoral neck fracture (fragility hip fracture), admitted to orthopaedic and orthogeriatric wards | CAM, systematic review of nursing staff notes | 133 | 514 | 83.1 |
| 059 | David M. Edelstein, 2004 ^69^ | M | USA | Non-pathologic femoral neck/ intertrochanteric fracture | Review of hospital chart notes, patient interview using DSM criteria, by an experienced geriatrician | 47 | 874 | unspecified |
| 063 | Emilija Dubljanin Raspopovic, 2015 ^70^ | M | Serbia | Acute hip fracture | CAM | 43 | 344 | 78.2 |
| 065 | Kannayiram Alagiakrishnan, 2009 ^71^ | M | Canada | Medical teaching units | Confirmed by geriatric medicine specialists using CAM | 20 | 132 | unspecified |
| 066 | Hochang B. Lee, 2017 ^72^ | M | USA | Hip fracture repair | CAM + MMSE | 150 | 466 | 80.8 |
| 069 | Matias Gonzalez, 2009 ^73^ | M | Chile | General medical ward | Likely Spanish CAM (information from caregiver and patients) | 192 | 542 | 77.9 |
| 070 | Malaz Boustani, 2010 ^74^ | M | USA | Inpatient general medicine service | CAM (patient examination, chart investigation, nurse and/or family member interview) | 163 | 424 | unspecified |
| 072 | Francisco J. Noriega, 2015 ^75^ | M | Spain | Cardiology department (Coronary care unit/ cardiology ward) for an acute cardiac condition (direct urgent admission) | CAM, CAM-ICU (for intubated patients) | 35 | 203 | 81.6 |
| 073 | Michael C. Large, 2013 ^76^ | M | USA | Radical cystectomy | CAM | 14 | 49 | unspecified |
| 074 | Maciej Bagienski, 2017 ^77^ | M | Poland | Patients undergoing TAVI, diagnosed with symptomatic severe AS, high surgical risk | Chart-based delirium identification instrument (CHART - DEL) | 29 | 141 | unspecified |
| 076 | Giovanni Falsini, 2017 ^78^ | M | Italy | Cardiac intensive care unit patients (orotracheal intubation excluded, non-invasive ventilation included) | RASS, CAM | 111 | 726 | 79.1 |
| 078 | Nadya Kagansky, 2004 ^79^ | M | Israel | Orthopaedic unit with hip fracture (excluding: patients with conservative treatment of hip fracture) | CAM, DRS | 12 | 102 | 82.5 |
| 080 | Martin G. Kat, 2011 ^80^ | M | Netherlands | Hip-fracture patients at intermediate/ high risk for delirium | DSM-IV, DRS-98 | 74 | 603 | 77.9 |
| 081 | Willem A van Eijsden, 2015 ^81^ | M, NH | Netherlands | Critical limb ischemia undergoing surgery (excluding: hospital stay <2 days) | Amphia risk score for delirium, DOSS, physician diagnosis of delirium based on DSM-IV | 29 | 92 | unspecified |
| 082 | ST Pendlebury, 2015 ^16^ | M | UK | All consecutive admissions to a single team (no exclusion criteria) | CAM, cognitive test (MMSE or AMTS), DSM-IV by physician | 95 | 308 | 81 |
| 084 | Giuseppe Bellelli, 2008 ^82^ | M, NH | Italy | Consecutively and newly discharged from RACU | CAM | Varies with outcome measured | Varies with outcome measured | unspecified |
| 085 | Rebecca Mitchell, 2017 ^83^ | M | Australia | Sustained a hip fracture | ICD-10-AM classification of F05, F05.0-F05.1, and F05.8-F05.9 in any of 50 diagnostic fields | 4065 | 27888 | unspecified |
| 087 | Meng-Chang Tsai, 2012 ^84^ | M | Taiwan | Inpatients seen in psychiatric consultation | Likely DSM-IV physician diagnosis | 172 | 429 | unspecified |
| 090 | J. W Raats, 2015 ^85^ | M | Netherlands | Colon, rectal cancer or diverticulitis surgery, admitted to department of gastrointestinal surgery | Shortened version (13 items) DOSS | 23 | 111 | unspecified |
| 091 | Paolo Mazzola, 2015 ^86^ | M | Italy | Acute care Orthogeriatric Unit (OGU), patients hip fracture and > 1 of these pre-fracture conditions: lack of social support, comorbidity, multiple drug treatment, use of oral anticoagulants | DSM-IV-TR by geriatrician, retrospective analysis of delirium occurred for some patients (identification of 10 key words associated with delirium in medical and nursing daily notes). | 135 | 275 | 89.4 |
| 095 | Markus F. Luger, 2014 ^87^ | M, D | Austria | Acute hip fracture surgery (either general or regional anaesthesia) | Entry of physician diagnosed delirium (DSM-IV criteria) in the chart | 18 | 329 | unspecified |
| 096 | Douglas L. Leslie, 2005 ^88^ | M | USA | 3 non-intensive care general medical units, intermediate/ high risk for delirium | CAM | 115 | 919 | 80 |
| 097 | Robbert C. Bakker, 2012 ^89^ | M | Netherlands | Cardiac surgery (deep hypothermia circulatory arrest, emergency procedure excluded) | Likely using CAM-ICU, in medical and nursing records | 63 | 201 | 76.1 |
| 100 | Monidipa Dasgupta, 2014 ^90^ | M, NH | England | General medicine in-patient teaching units (excluding: transfer to non-medical ICU or surgical service) | Chart audit tool (documentation of key delirium symptoms), brief mental status screening using SPMSQ, research assistant asking family members, caregivers or staff interview or chart records, structured interview, CAM criteria, MDAS, IQCODE | 355 | 1235 | 82.6 |
| 102 | Joseph Francis, 1992 ^91^ | M | USA | General medical service | Applying information from the entire hospital evaluation to DSM-III-R criteria | 34 | 180 | unspecified |
| 104 | Dimitrios Adamis, 2006 ^92^ | M, NH | UK | Elderly care unit | CAM, DRS | 33 | 94 | 82.8 |
| 105 | Jelle W. Raats, 2015 ^93^ | M | Netherlands | Elective surgery for colorectal cancer or Abdominal Aortic Aneurysm (excluding: emergency surgery) | Scored prospectively using DOSS (shortened version with 13 items), geriatrician consult, DSM-IV used to confirm diagnosis | 35 | 232 | unspecified |
| 107 | Leslie S P Eide, 2016 ^94^ | M | Norway | Elective treatment for severe aortic stenosis with SAVR or TAVI | CAM, medical, nursing and physiotherapists' reports from previous 24-hours | 76 | 136 | 83.5 |
| 110 | Rosalind Ramsay, 1991 ^95^ | M | UK | Acute geriatric admissions and specifically referred for rehabilitation | Using DSM-III-R after completion of the questionnaires and interview within 7 working days of admission | 22 | 88 | unspecified |
| 112 | Rungnirand Praditsuwan, 2013 ^96^ | M | Thailand | General medical wards | Geriatrician diagnosed delirium according DSM-IV criteria | 110 | 225 | 78.0 |
| 113 | Koji Sato, 2017 ^97^ | M | Japan | Non-surgical cardiac patients admitted to ICU or intensive cardiac care unit (excluding: mechanically ventilated patients) | CAM-ICU by well-trained nurses | 35 | 105 | unspecified |
| 116 | Martin G. Cole, 2008 ^98^ | M | Canada | Admitted to medical or geriatric services (excluding: ICU, cardiac monitoring unit) | CAM, Chart, family and nursing staff used to complete CAM, delirium diagnosed if DSM-III-R criteria met, Delirium Index used at 8 weeks | 115 | 210 | unspecified |
| 117 | Kathy H. Whittamore, 2014 ^99^ | M, NH | UK | Unplanned admission to one of the following wards: 3 acute geriatric ward, 2 trauma orthopaedic, 7 general medical | DRS-R-98 diagnosed delirium | 107 | 247 | unspecified |
| 118 | Allan Gottschalk, 2015 ^14^ | M | USA | Undergoing hip fracture repair | CAM by attending geriatrician/ trained research nurse | 151 | 459 | 81.3 |
| 120 | Kenneth Rockwood, 1999 ^100^ | M, D | Canada | Admitted to the general medicine service | DSM-IV operationalized with clinical judgement, supplemented by MMSE, Delirium Rating Scale and rating of illness severity | 38 | 203 | unspecified |
| 123 | Meng-Chang Tsai, 2012 ^101^ | M | Taiwan | Psychiatric-consulted inpatients | DSM-4th edition text revision criteria | 172 | 614 | 74.7 |
| 124 | Shaun O' Keeffe, 1997 ^102^ | M | Ireland | Acute care geriatric unit | Semi-structured interview protocol, Delirium Assessment Scale to elicit the presence and severity of DSM-3 criteria (except criterion 5) | 94 | 225 | unspecified |
| 125 | Sharon K. Inouye, 2016 ^103^ | M | USA | Surgical patients: lumbar cervical/ sacral laminectomy, total hip/knee replacement, open abdominal aortic aneurysm repair, lower extremity arterial bypass surgery, and open/ laparoscopic colectomy | Brief cognitive testing, Delirium Symptom Interview, family and nurse interviews. CAM, validated chart review method used. | 134 | 560 | 76.7 |
| 127 | Mitsunaga Iwata, 2006 ^104^ | M | Japan | Admitted to the acute care setting | DSM-IV | 44 | 403 | 88.7 |
| 129 | Shanmugam Uthamalingam, 2011 ^105^ | M, NH | USA | Diagnosis of acute decompensated heart failure | CAM (data from physician, nursing, physical and occupational therapist medical records, abbreviated mental test, clinical signs and symptoms etc.) | 151 | 883 | 79 |
| 131 | Aik Haw Tan, 2015 ^12^ | M | New Zealand | One medical ward, one orthopaedic ward and one assessment, treatment and rehabilitation ward | CAM, MMSE | 28 | 250 | unspecified |
| 133 | Sue. E Levkoff, 1992 ^106^ | M | USA | Medical and surgical setting | Delirium Symptom Interview, medical charts, DSM-III etc. | 125 | 325 | 81.4 |
| 134 | Ozge Duman Atilla, 2014 ^107^ | M | Turkey | Admission via ED | CAM (shortened version) | 49 | 693 | 76 |
| 135 | Melanie Dani, 2018 ^108^ | M | UK | Acute medical unit | Evaluated by trained psychiatrist, information from MMSE, CAM, ward staff and medical chart | 73 | 710 | 83.1 |
| 137 | Carlos Jorge-Ripper, 2017 ^109^ | M | Unspecified | General internal medicine unit | CAM | 83 | 119 | 75.8 |

Characteristics of the 71 included studies. M: mortality, NH: nursing home, D: dementia. CAM: Confusion Assessment Method, DSM: Diagnostic and Statistical Manual of Mental Disorders, MMSE: Mini-Mental State Examination, DRS-R-98: Delirium Rating Scale-Revised-98, DOSS: Delirium Observation Screening Scale, OBS: Organic Brain Syndrome, DOS: Delirium Observation Scale, MDAS: Memorial Delirium Assessment Scale


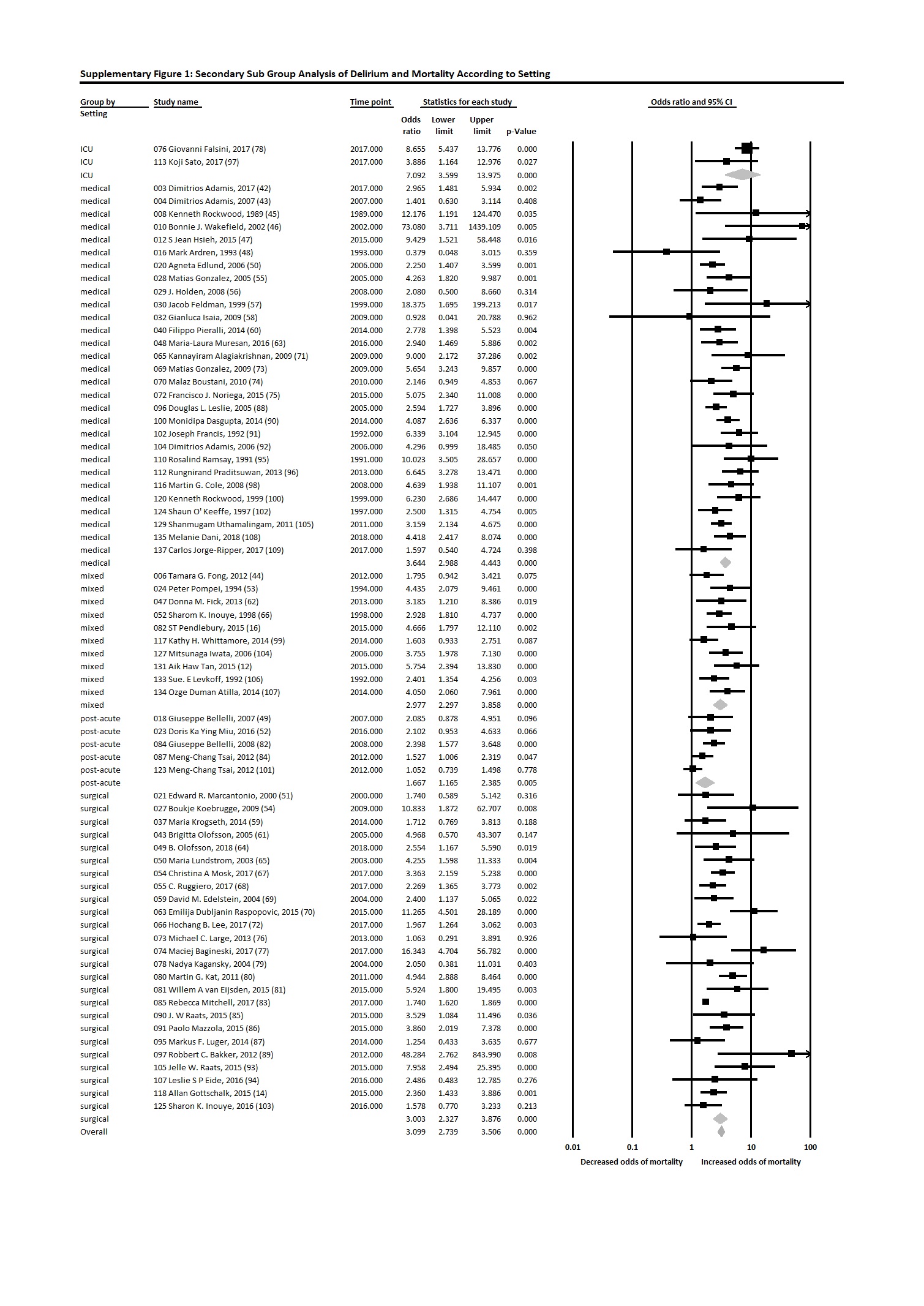


Supplementary Figure S1 Analysis of unadjusted data from 71 studies, sub-grouped into setting (ICU, medical, post-acute, surgical or mixed). Odds Ratio of >1 indicates greater odds of mortality in patients who experienced delirium. Square size corresponds to study size and are proportional to each other within the subgroup.
